# Supplementary material for: Transcriptome profiling and characterization of peritoneal metastasis ovarian cancer xenografts in humanized mice
Source: Sci Rep. 2024 May 24;14:11894. doi: 10.1038/s41598-024-60501-z (PMC11126626; doi:10.1038/s41598-024-60501-z)
Supplement: Supplementary file 1 — Supplementary Legends. [file 41598_2024_60501_MOESM1_ESM.docx]

**SUPPLEMENTARY FIGURE LEGENDS**

**Supplementary Figure 1. Growth of ovarian cancer cells in the peritoneal cavity of Hu-mice using bioluminescence imaging (BLI).**

(A) In the second animal experiment, in vivo BLI were obtained repeatedly up to 6-8 weeks following the IP injection of OVCAR-3 Luc and SKOV-3 Luc cells into Hu-mice. The escalation in BLI intensity of photons over time indicated the growth of (B) OVCAR-3 Luc and (C) SKOV-3 Luc tumors.

**Supplementary Figure 2. Body weight changes in Hu-mice bearing SKOV-3 Luc xenograft tumors treated with PBS or pembrolizumab.**

During the periods of pembrolizumab administration, the body weights of the Hu-mice remained relatively stable with no notable alterations.

**Supplementary Figure 3. Histological examination of SKOV-3 Luc xenograft tumors in Hu-mice treated with PBS or pembrolizumab.**

Histopathological examination of SKOV-3 Luc xenografts in Hu-mice was performed by H&E staining. (**A**) Tumor tissues were resected from the intraperitoneal cavity of Hu-mice at the experimental endpoint. (**B**) Representative images show SKOV-3 Luc-derived tumor tissues.

**Supplementary Figure 4. Characterization of human lymphocytes in SKOV-3 Luc tumor-bearing Hu-mice treated with PBS or pembrolizumab.**

Frequencies of human CD4^+^ T cells, CD8^+^ T cells, and CD19^+^ B cells in the blood were assessed using flow cytometry. Data from mice treated with either PBS or pembrolizumab were analyzed and compared between two groups: ND_BLI, denoting cases with no BLI detected at the end of the experiment, and PS_BLI, indicating cases with constant or increasing BLI exposure by the end of the experiment. Statistical significance was determined using unpaired t-test (* P < 0.05, ** P <0.01, *** P < 0.001).

**Supplementary Figure 5. Immune cell composition in SKOV-3 Luc xenograft tumors in Hu-mice treated with PBS or pembrolizumab.**

The bar chart illustrates the relative abundance ratio of 22 immune cell subsets in tumors from the PBS and pembrolizumab groups. The data was obtained through CIBERSORTx using the LM22 immune cell gene signature, and the visualization was automatically created using the CIBERSORTx web server.

**Supplementary Figure 6. Enrichment plots from gene set enrichment analysis (GSEA) of the ten gene signatures.**

Representative gene signatures were significantly enriched in the SKOV-3 Luc-derived tumors of Hu-mice treated with pembrolizumab vs. PBS control (FDR < 0.25). The enrichment score (ES) is shown on the y-axis, while the x-axis represents genes (represented as vertical black lines) found in the gene sets. All gene sets were selected from the molecular signatures database (MSigDB), specifically from categories C2, C5, C7, C8, or H.

**Supplementary Figure 7. Gating strategy for flow cytometry.**

(A) Representative plots showing the gating strategy employed in the flow cytometry analysis of human leukocyte cell populations in Fig. 2 and 3 using the FlowJo software. Single cells were first gated on a forward scatter-area (FSC-A)/height (FSC-H), followed by gating for live cells and human CD45^+^ cells. Human CD56^+^, CD11b^+^, CD19^+^, and CD4^+^/CD8^+^ cells were derived from the region gated for human CD45^+^. (B) Frequencies of human CD4^+^ T cells, CD8^+^ T cells, and CD19^+^ B cells expressing PD-1 or not in the blood of PBS and pembrolizumab groups were evaluated using flow cytometry. Initially, leucocytes were isolated by gating on FSC-A and forward scatter-area (SSC-A), which was then followed by further gating to identify single cells and live human CD45^+^ cells. From the population of CD45^+^ gated cells, human CD19^+^PD-1^+^ B cells, CD3^+^CD4^+^PD-1^+^, and CD3^+^CD8^+^PD-1^+^ T cells were subsequently distinguished.
