# Supplementary figures and images for: Transcriptome profiling and characterization of peritoneal metastasis ovarian cancer xenografts in humanized mice
Source: Sci Rep. 2024 May 24;14:11894. doi: 10.1038/s41598-024-60501-z (PMC11126626; doi:10.1038/s41598-024-60501-z)

**A**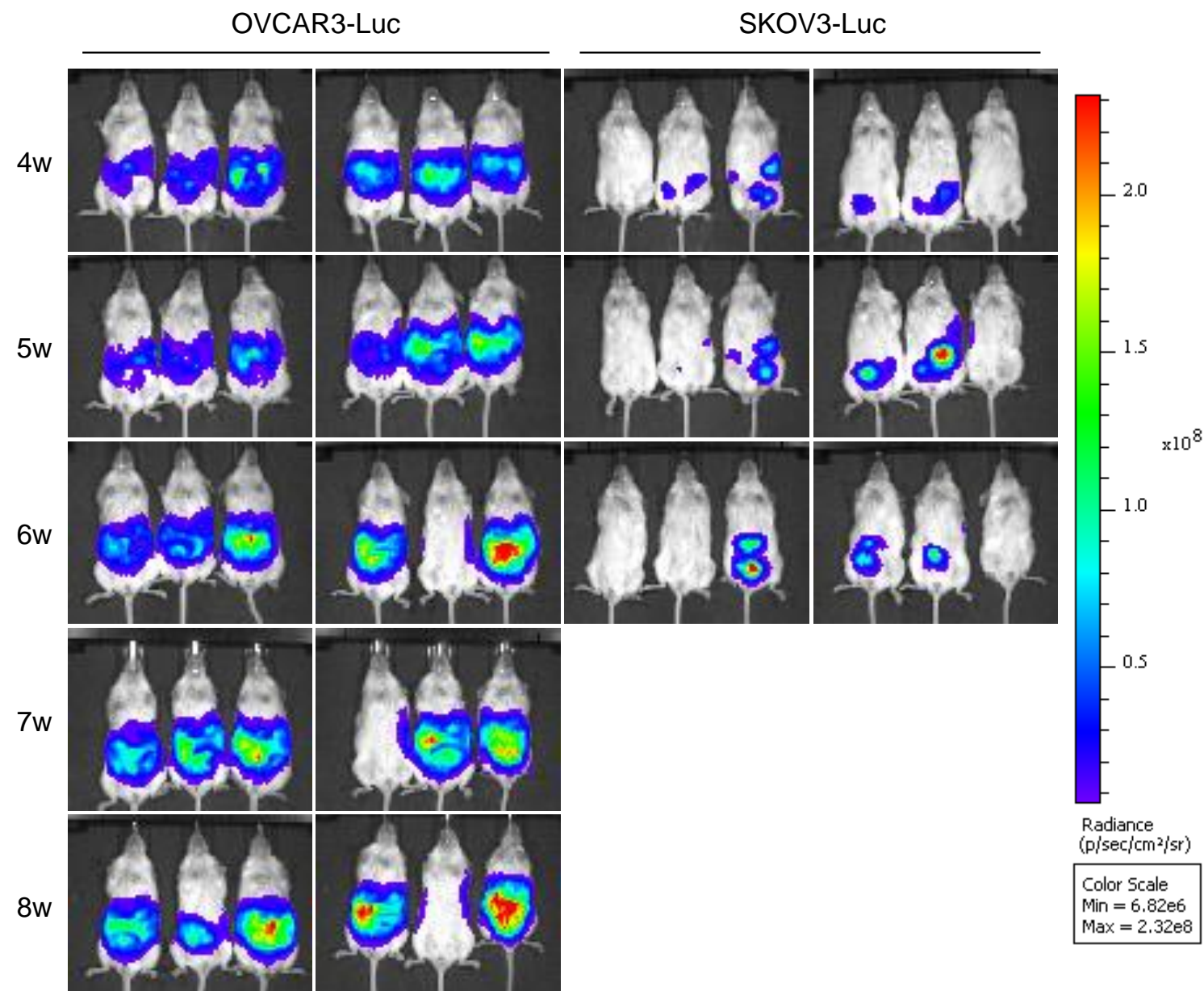**B**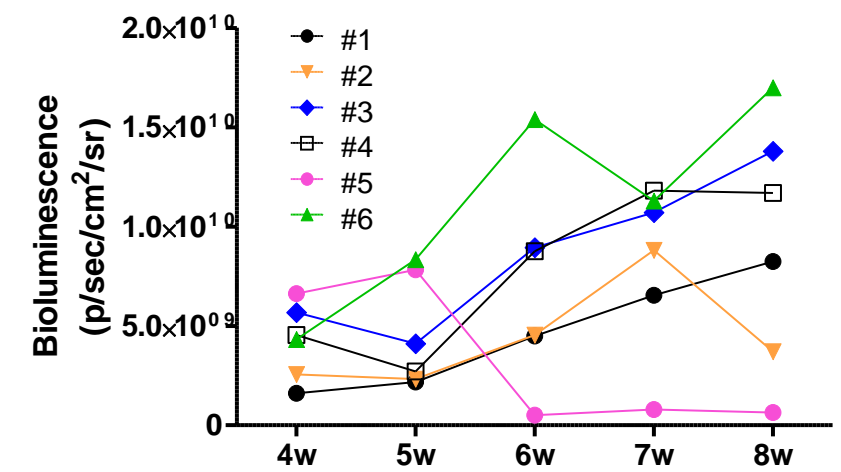**C**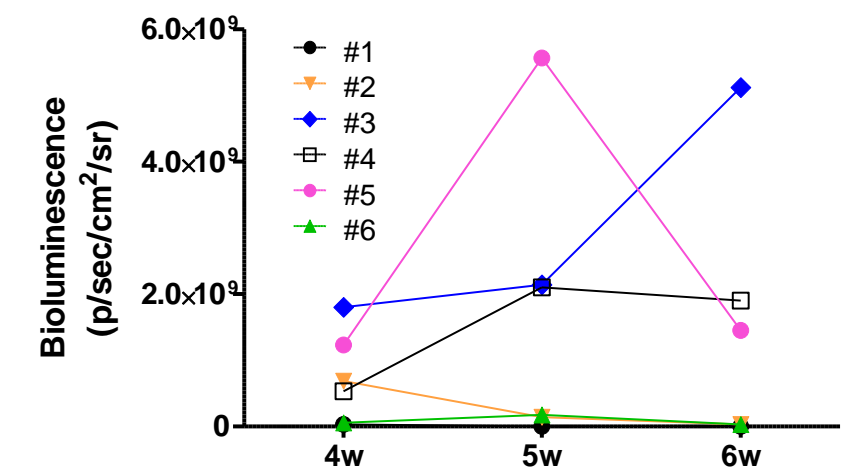

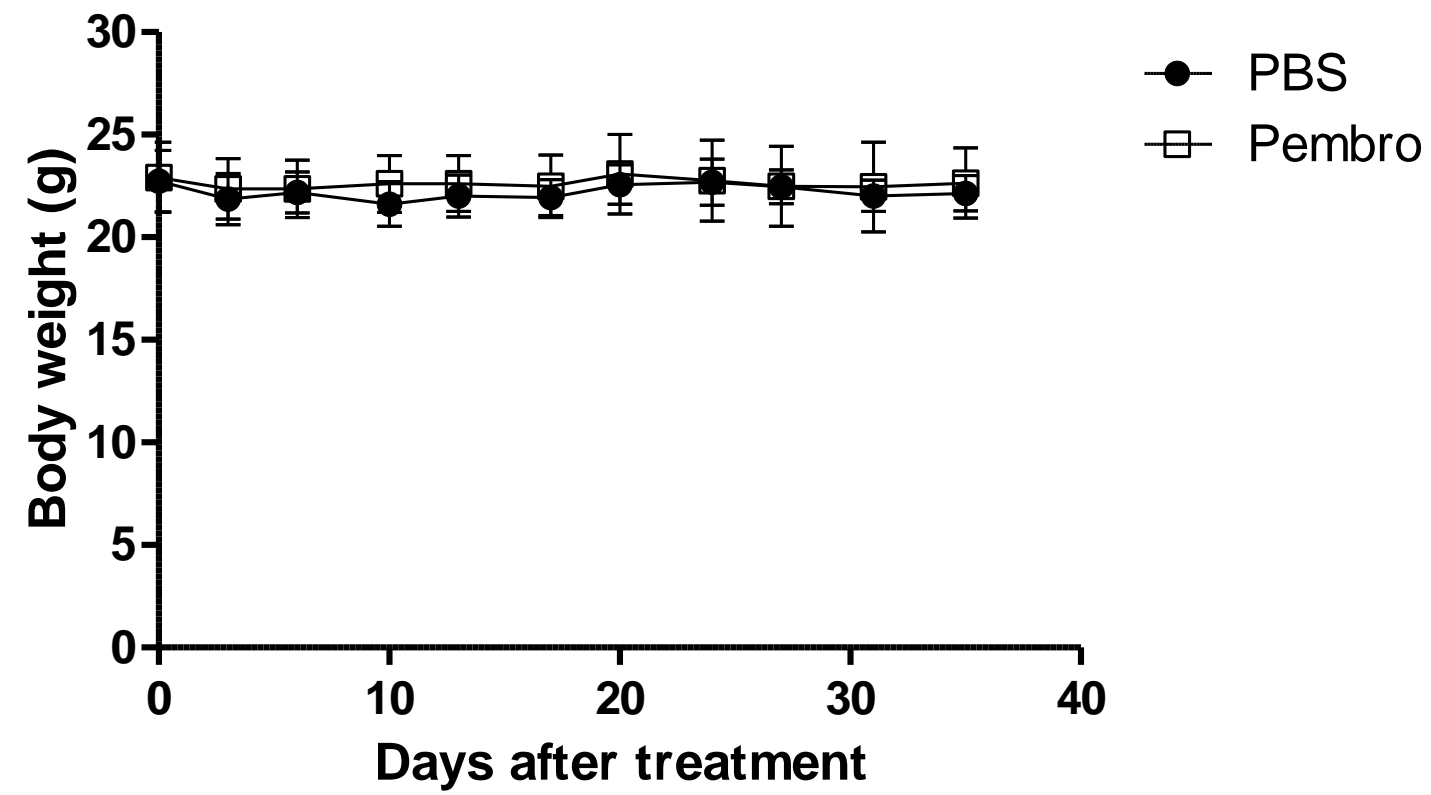

**A**

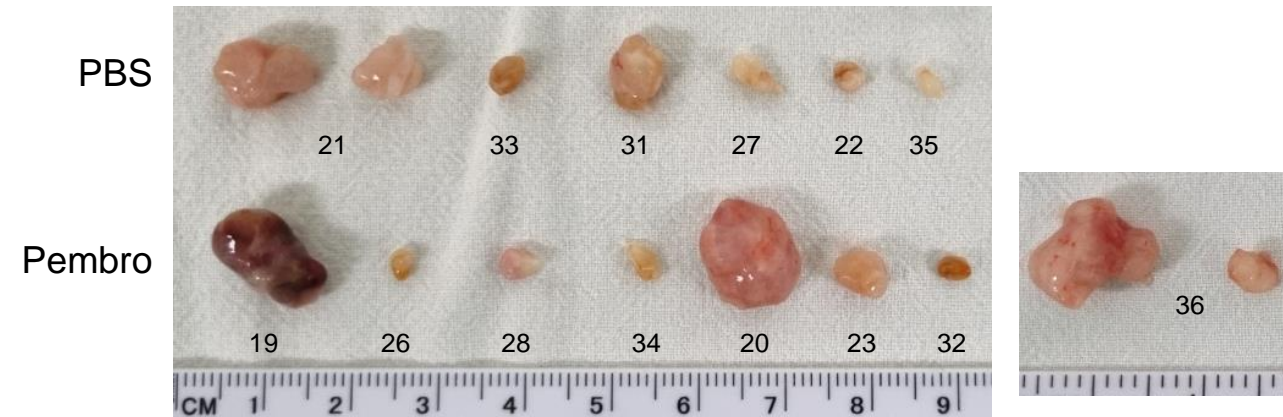

**B**

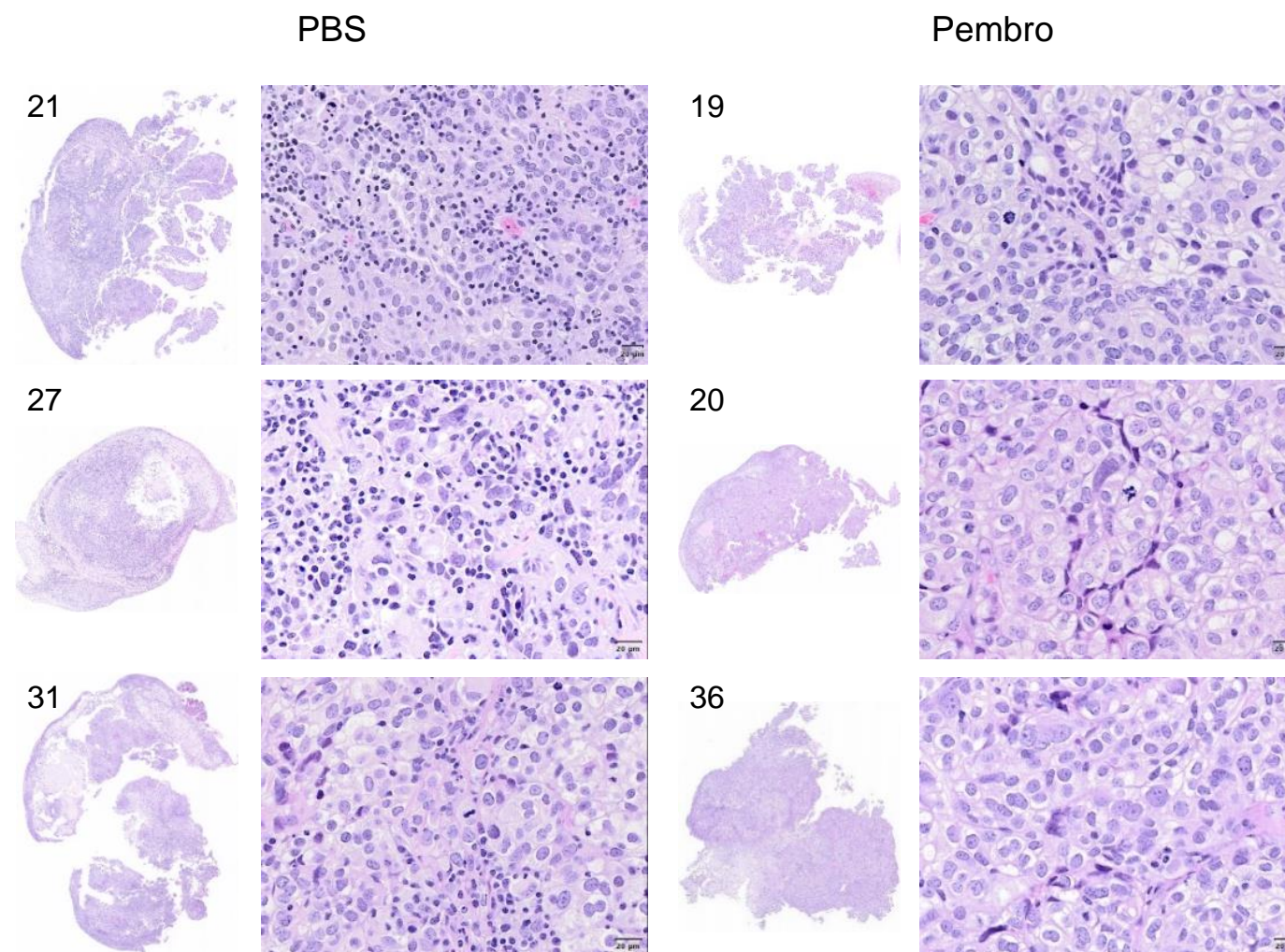

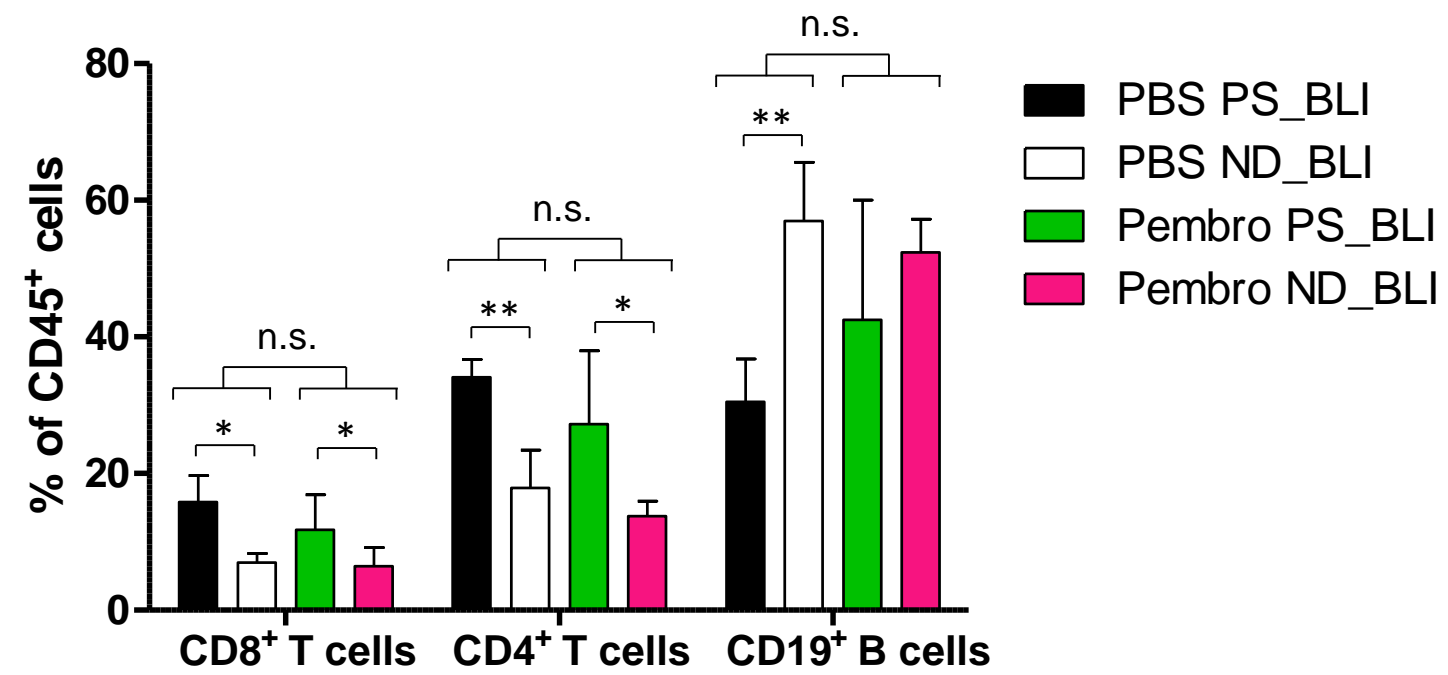

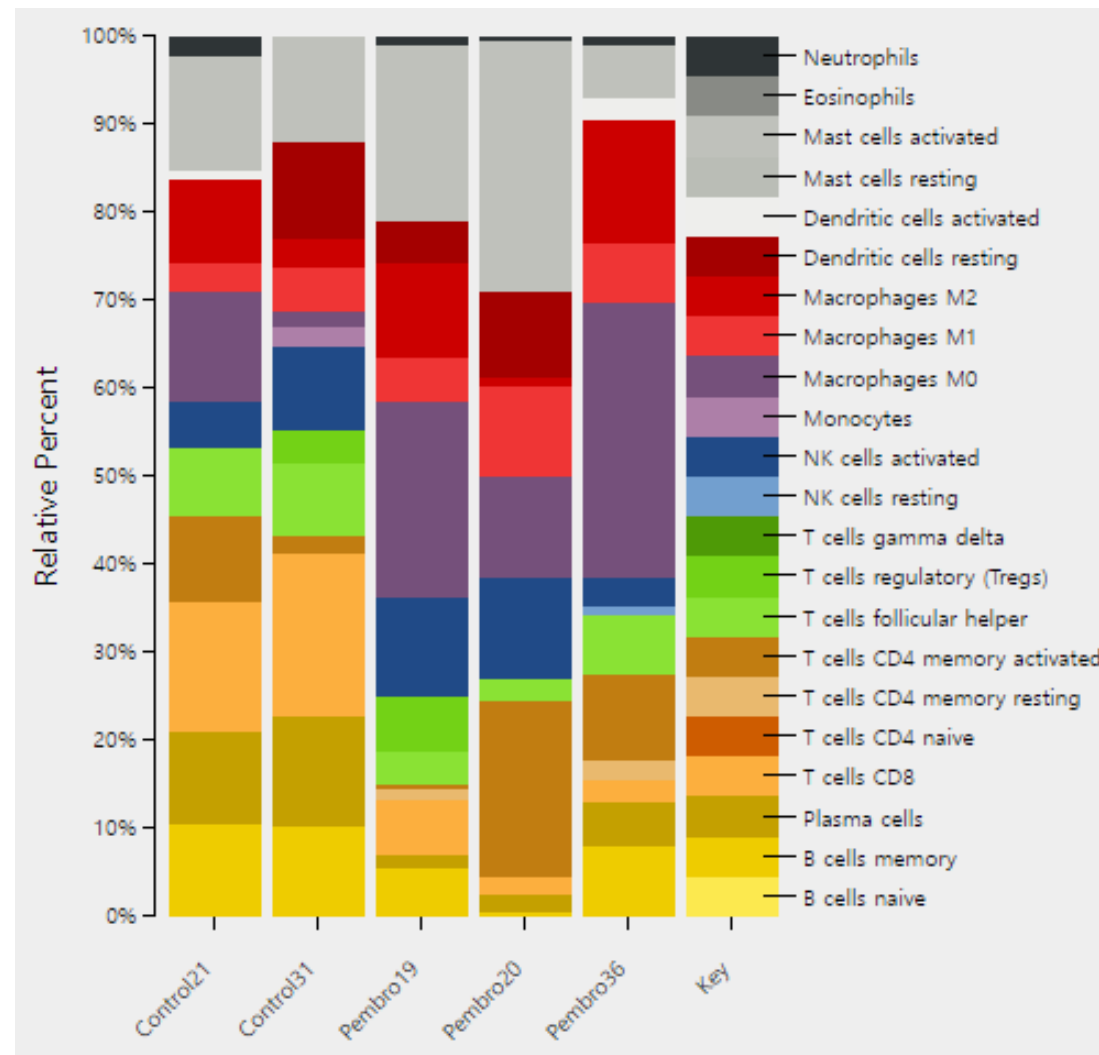

A

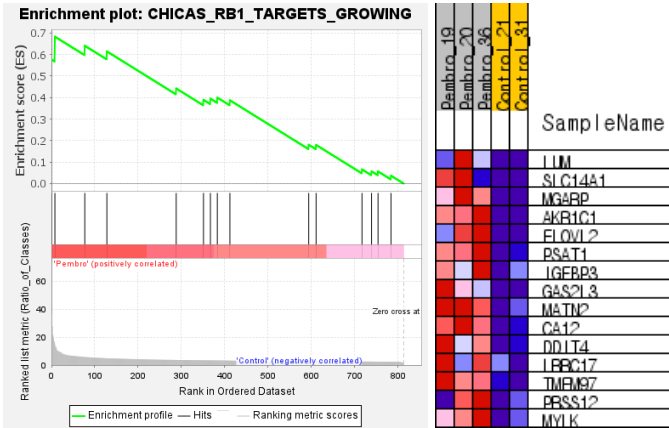

B

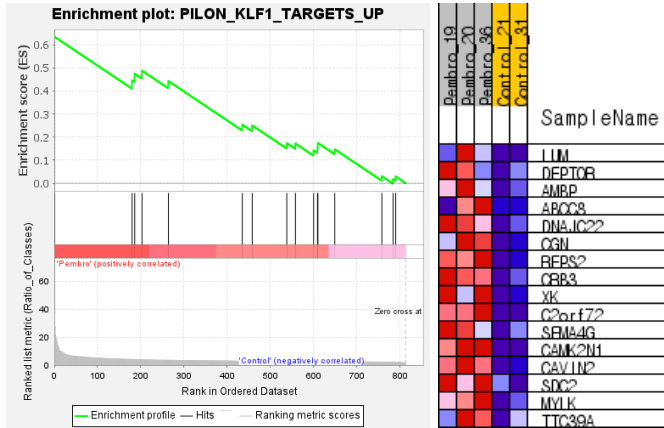

C

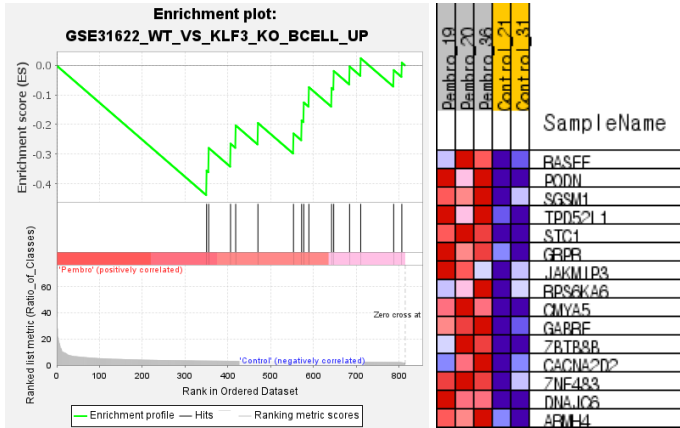

D

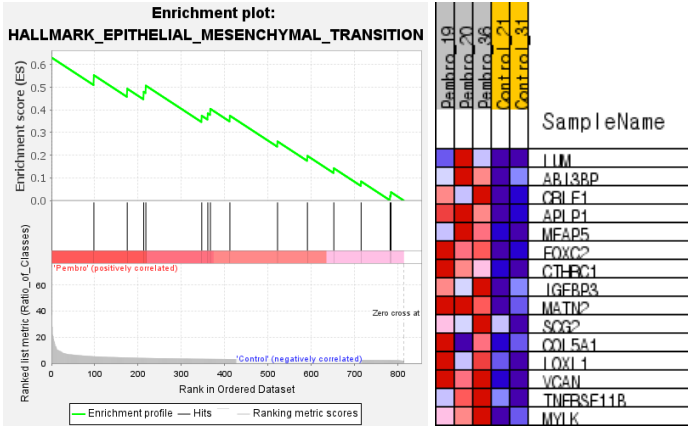

E

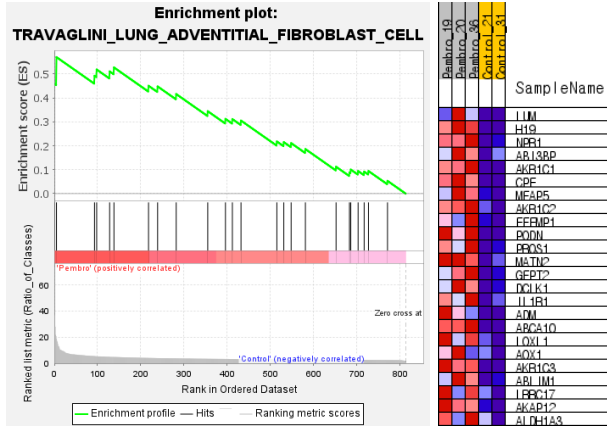

F

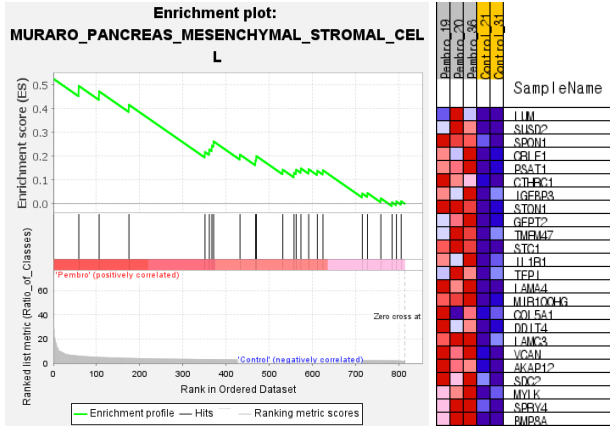

G

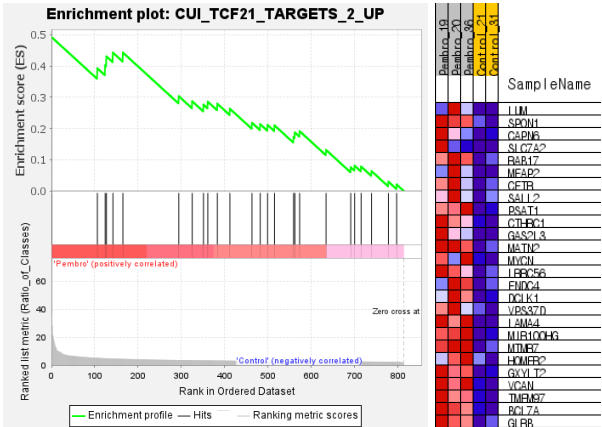

H

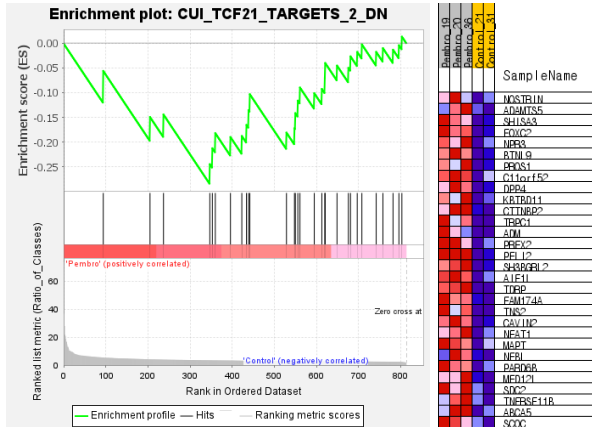

I

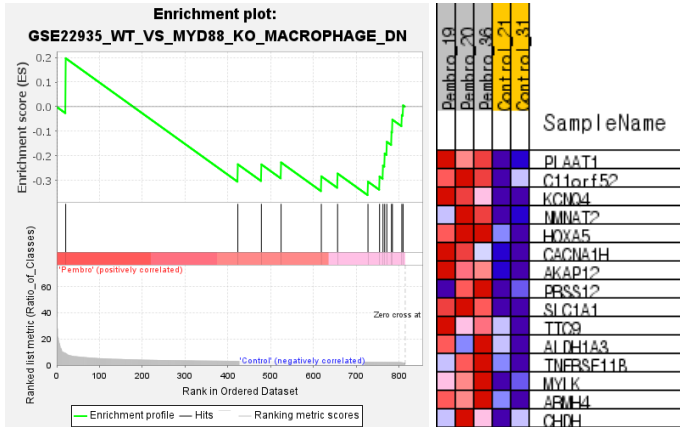

J

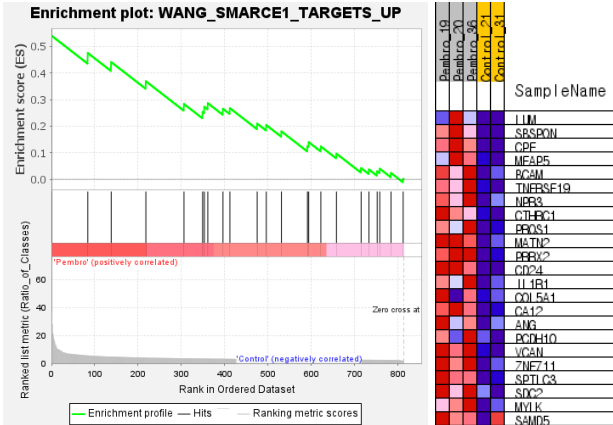

**A**

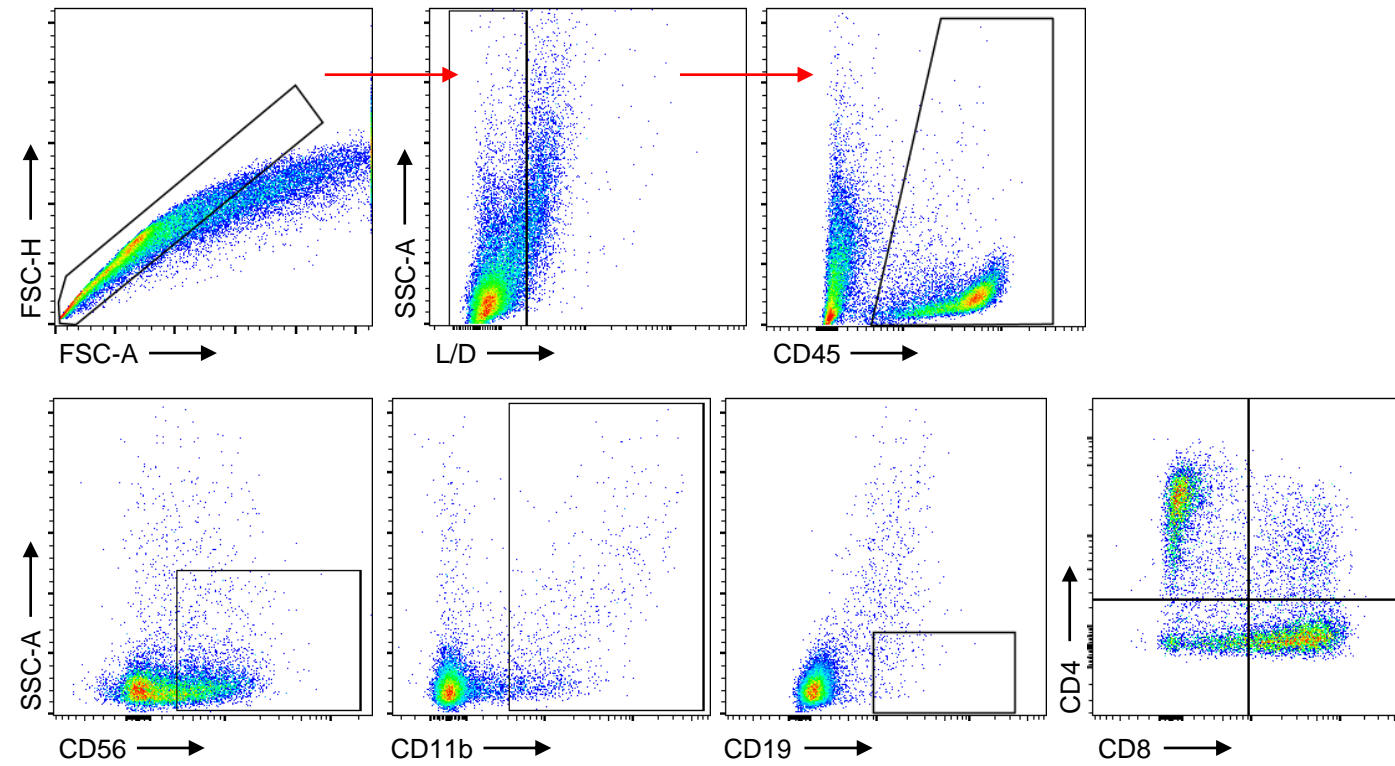

**B**

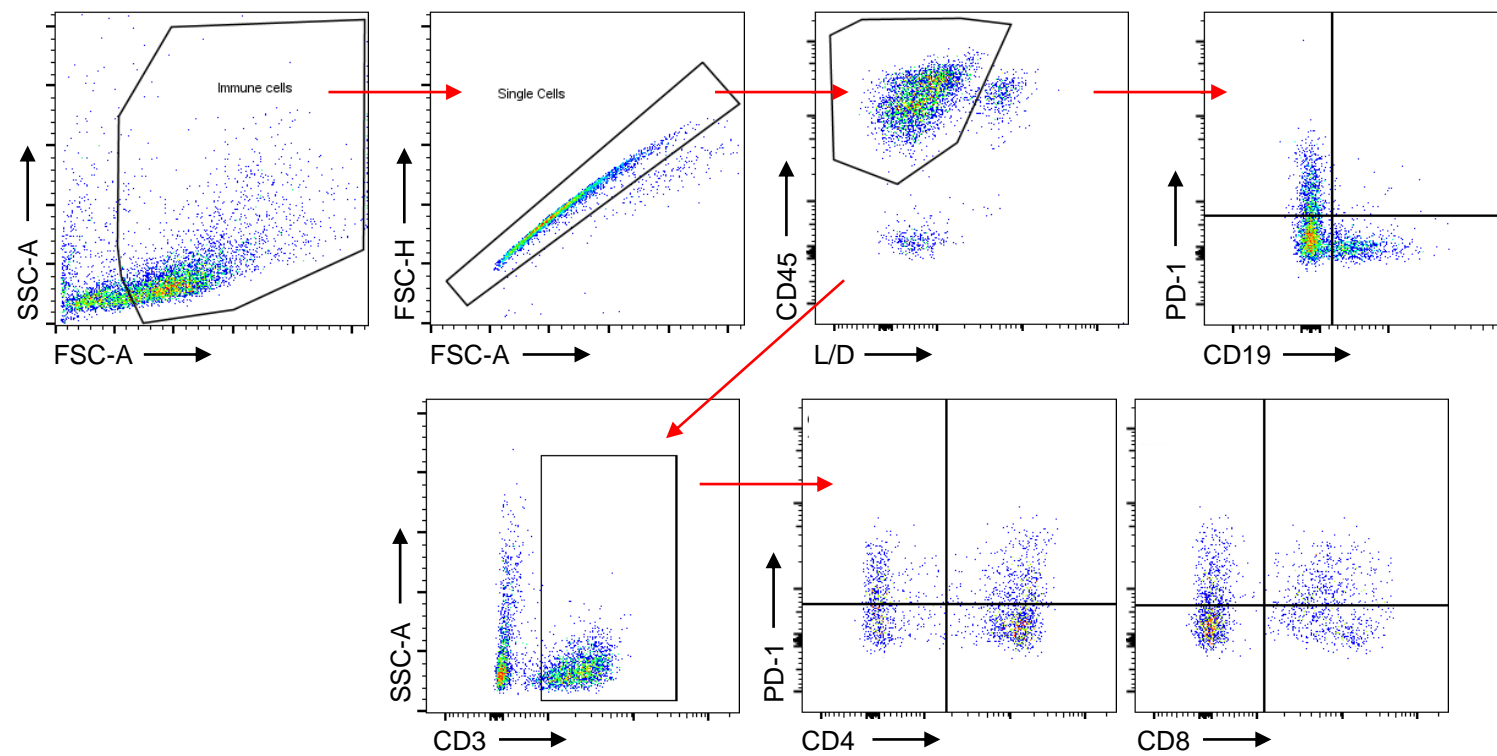

Supplement: Supplementary file 2 — Supplementary Figures. [file 41598_2024_60501_MOESM2_ESM.pdf]
